# Air Pollution and Radiation Monitoring in Collective Protection Facilities

**DOI:** 10.3390/s23020706

**Published:** 2023-01-08

**Authors:** Angelika Monika Kołacz, Monika Wiśnik-Sawka, Mirosław Maziejuk, Marek Natora, Władyslaw Harmata, Paweł Rytel, Dorota Gajda

**Affiliations:** 1 Military Institute of Chemistry and Radiometry, al. gen. A. Chruściela “Montera” 105, 00-910 Warsaw, Poland; 2 Faculty of Advanced Technologies and Chemistry, Military University of Technology, ul. gen. S. Kaliskiego 2, 00-908 Warsaw, Poland

**Keywords:** ion mobility spectrometry (IMS), chemical warfare agent (CWA), toxic chemical (TIC), radiation hazards

## Abstract

It has become increasingly important to monitor environment contamination by such chemicals as chemical warfare agents (CWAs) and industrial toxic chemicals (TICs), as well as radiation hazards around and inside collective protection facilities. This is especially important given the increased risk of terrorist or military attacks. The Military Institute of Chemistry and Radiometry (MICR) has constructed and developed the ALERT device for the effective monitoring of these threats. This device uses sensors that detect chemical and radiological contaminations in the air. The CWA detector is an ion mobility spectrometer, TICs are detected by electrochemical sensors, and radiation hazards are detected via Geiger–Muller tubes. The system was designed to protect the crew from contamination. When chemical or radioactive contamination is detected at the air inlet for the shelter, air filtration through a carbon filter is activated. At this time, the air test procedure at the filter outlet is started to test the condition of the filter on an ongoing basis. After detecting contamination at the filter outlet, the system turns off the air pumping and the service can start the procedure of replacing the damaged carbon filter. This paper presents the results of laboratory testing of the ALERT gas alarm detector, which showed high measurements for important parameters, including sensitivity, repeatability, accuracy, and speed.

## 1. Introduction

Initiator monitoring can cause serious threats, e.g., due to the usage of chemical, biological, radiological, and nuclear (CBRN) agents, which is especially important due to increasing risks of terrorism and war. There is a demand for providing safety systems for the recognition and continuous air monitoring of these harmful factors as well as protection against them. Therefore, it has become necessary to equip buildings, vehicles, and public utility facilities with stationary contamination and radiation recognition systems, so that continuous air monitoring and protection is carried out at important venues [1].

Contamination recognition is an activity aimed at determining the usage of weapons of mass destruction (WMD), releases other than attack (ROTA) events, or the presence of radioactive substances or biological or chemical agents. The contamination identification process includes detection, preliminary and detailed contamination identification, and observation. Detectors used in this process is divided into warning (detect-to-warn) and analytical (detect-to-treat) applications [2,3].

Collective protection facilities are stationary, mobile, transportable, or ad hoc (temporary), and are designed to protect people. They are specially prepared and adapted facilities designed to protect people and equipment from the effects of conventional weapons, weapons of mass destruction, and toxic industrial chemical (TIC) contamination. Among these facilities, shelters and hideouts can be distinguished, but also vehicles, vessels, etc. [4,5].

Collective protection facilities of the stationary or mobile type should be prepared and adapted to protect people and equipment from the effects of weapons of mass destruction as well as TICs. This may include the installation of high-performance filtering system equipped with filter canisters to protect the respiratory system, which is the main gateway for the entry of gaseous and aerosol contaminants into the human body. Highly toxic substances can be removed from the air stream using various functional materials, such as high-efficiency air filters, molecular sieves, bentonites, or porous materials such as activated carbon [6,7,8].

An Important aspect of safety is the duration of the protective action of modern filter absorbers and the need for their safe replacement. This time-period is limited and closely related to the concentration of contaminants. It is worth mentioning that filter absorbers do not neutralize toxic substances, but only filter and adsorb them. Therefore, technical conditions have been developed for each type of filter absorber; they contain operational and protective requirements, including permissible air flow resistance, sorption capacity, and protection time for selected substances, taking into account their type (acidic, alkaline, or organic/inorganic) [9,10,11].

This paper is focused on using a multifunctional device, ALERT, for continuous air pollution, radiation monitoring, and the monitoring of filter quality.

After detecting pollution in the intake of air, ALERT tests the inner air of the shelter (after the carbon filter).

The ALERT gas alarm detector is a device designed by the Military Institute of Chemistry and Radiometry (MICR). It is a device capable of measuring CWAs, TICs, and ionizing radiation. An integral part of this device is the ion mobility spectrometer with drift tube (DT IMS) for detecting CWA contamination, TICs detection by EC4-CLO2-5ppm and SGX-7NH3 electrochemical sensors (Angst+Pfister), and ionizing radiation detection by externally connected dosimetry probes, (Polon Alfa). A multifunctional device such as ALERT is hard to compare with commercially available devices. Its uniqueness lies in the fact that all three types of detection are cumulated in one device and, this device also controlling the air filter, then the crew are protected against the pollutions. The research we have conducted proves that the reaction time to the contamination of CWAs, TICs, and ionizing radiation is very fast and is at most 30 s. The ALERT gas alarm device, i.e., the chemical pollution detector, has a patent, thanks to which it is installed and used on ships of the Polish Navy (patent number P.348124). Taking into account the measurement capabilities of the ALERT gas alarm device, it is possible to use it in civilian collective protection facilities.

## 2. Materials and Methods

### 2.1. ALERT: General Description

The stationary ALERT continuous operation system is equipped with a chemical contamination detector for detecting such chemicals as CWAs and TICs, as well as with radiation detectors, which measure elevated radiation background. This gas alarm device performs system diagnostics permanently and has the ability to acquire data on contamination and transfer these data to the system. It can also automatically activate the filtering device after the detection of contamination. It can be utilized for interior and exterior operations [12,13].

The ALERT device is designed to provide security and early warnings of threats. This device can be part of the equipment of fixed and mobile collective protection facilities, as well as weapons and military technology (transporters and ships) [14,15].

**Figure 1:**
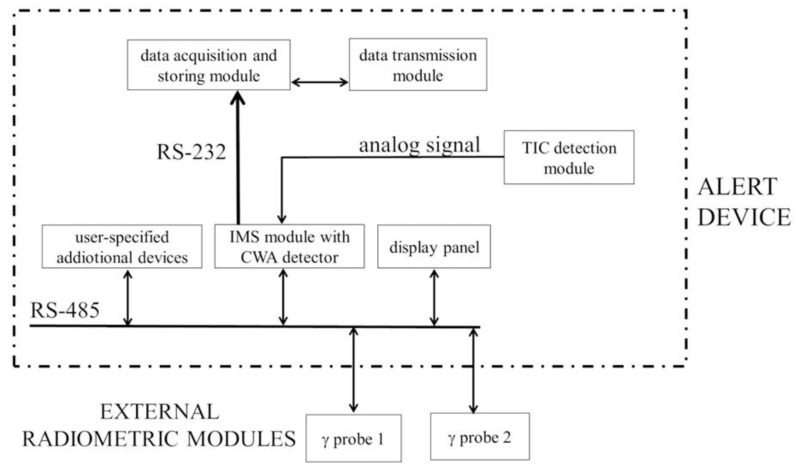
The block scheme of the ALERT device.

The scheme of communication of the particular modules with the system is shown in Figure 1.

ALERT consists of a small-size industrial computer with a flash disk with a capacity of several dozen gigabytes for acquiring and storing the obtained data. This data is used for informing the regional or national contamination analysis centers via radio, landline, or mobile phone. Data collected include the type of chemical contamination and its concentration, the gamma radiation dose rate, and the measurement time.

The ALERT device is equipped with an IMS module, which detects all CWAs, as well as a TIC module, which detects such TICs as hydrogen cyanide, chlorine, and phosgene. These modules are the internal part of the ALERT (as shown in Figure 1).

The elevated ionizing radiation background is measured by the two gamma radiation probes that are wired to ALERT. These two probes can trigger alarming signals for two dose rate thresholds. Depending on the probe type, neutron radiation might also be detected. ALERT is usually also equipped with ZR-1 and ZR-2 probes, but other ionizing radiation detectors can be used if they are compatible.

**Figure 2:**
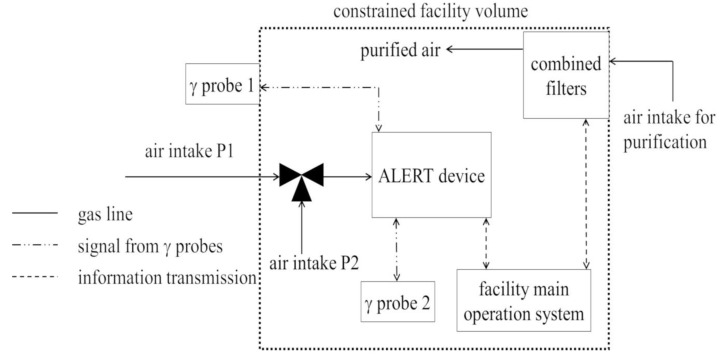
The scheme of a possible gas line option for the ALERT signaling device and the associated system location in the collective protection facility.

The scheme of possible gas line options for the ALERT gas alarm device and the location of the system is shown in Figure 2. An air sample is taken from the external shelter space (facility) by the P1 inlet. After detecting contamination in the air, the system switches a solenoid valve, and the air is taken from the space inside the shelter (P2 inlet) after carbon filter. In this way, we may control the conditions of the filters. If we detect pollutions after such a filter, we t subsequently begin to replace the filter.

### 2.2. Module Description

#### 2.2.1. IMS Module: CWAs Detection

**Figure 3:**
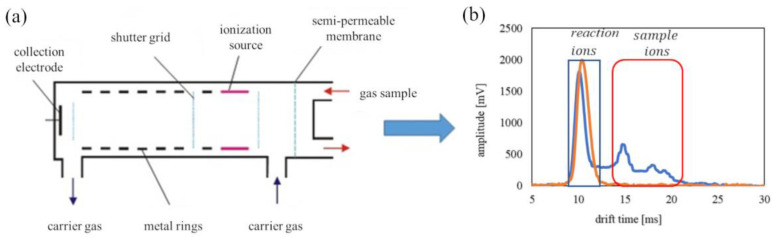
(**a**) IMS with the DT IMS detector principle of operation and (**b**) an exemplary drift time spectra for hydronium ions.

The ionization method is based on ion mobility spectrometry (IMS) and is utilized for measurement of CWA molecular ion drift in an electric field [16,17,18]. Based on the IMS method, many portable devices, such as CAM and its modified versions, and transportable devices, such as GID-2, GID-3, and ALERT, for contamination detection, have been designed and commissioned. The working principle is shown in Figure 3 [19,20].

This technique has many analytical applications. For example, it is used by police to detect prohibited materials such as drugs and explosives. The combination of IMS detectors with other techniques, such as gas chromatography (GC) or mass spectrometry (MS), are becoming more commonly used for the chemical analysis of many organic compounds [21,22,23,24].

The DT IMS detector is divided into two areas: the ionization zone, where ions are formed, and the drift zone, where ions are separated [25,26,27]. For this detector, the radioactive nickel isotope ^63^Ni (low beta decay) has been used as a source of ionizing radiation for the ionization of chemical molecules. Subsequently, ions created in the ionization zone are introduced into the drift zone by a dosing grid, which allows for a specific pack of ions to be introduced into the drift zone. The ion separation is based on the unique mobility of the ionized molecules in a low-intensity electric field (around 250 V/cm). The signal is generated by the collecting electrode. In this way, a drift time spectrum is obtained. The spectra shows the reaction ions, i.e., the hydronium and analytical ions, as monomeric ions (MH^+^(H_2_O)n) and dimeric (M_2_H^+^). An exemplary spectrum is shown in Figure 3 [28,29]. The ion mobility spectrometer enables chemical identification and quantification [30]. The identification of compounds is based on the ion mobility coefficient (K = (cm^2^V^−1^s^−1^)) described as

*K* = V_d_/E (1)

where V_d_ is the drift velocity (cm/s) and E is the intensity electric field (V/cm). K strictly depends on the pressure, P, and temperature, T, of the gas. To compare the obtained results, it is necessary to use the reduced mobility coefficient K_0_, which is a normalized K value for a temperature of 273 K and a pressure of 760 Torr. This correlation is shown in Equation (2) [31,32].

*K*_0_ = *K* (273 · *T*)/(*P* · 760) (2)

The part of the ALERT system responsible for detecting chemical substances consists of highly sensitive elements. The value of K_0_ for CWAs is strictly designed for such chemicals, and as such peaks are detected, the gas alarm device turns the alarm and filter switch on. The estimated reaction time of the signaling device to contamination ranges is several dozen seconds. The detection thresholds of particular CWAs are shown in Table 1.

The carrier gas is dehumidified and purified air. The sample flows (Figure 3) to the inlet in the IMS chamber but only before the semi-permeable membrane. The humidity is stopped, but the other chemical (also a CWA) is not; thus, we can detect CWAs easily.

If the sieve pack (dehumidifier) does not work properly, then we cannot detect sulfur mustard. Because of this, technical service is performed every two years.

TIC Module, the measurement of CWAs and some TICs range within a single ppm for Alert device.

The gas alarm device allows for the detection of such chemicals as molecular chlorine and ammonia. The use of an additional module allows for the detection of nitrogen oxide, nitrogen dioxide, sulfur dioxide, hydrogen chloride, hydrogen fluoride, carbon monoxide, and hydrogen sulfide [33,34].

For detecting chemicals from the TIC group, ALERT has been equipped with two gas electrochemical sensors (SGX Angst+Pfister).

One sensor is dedicated to chloride detection, the second to ammonia detection. Their reaction time, determined experimentally, is up to 30 s. The detection limits of these two sensors are shown in Table 2.

#### 2.2.2. Gamma Radiation Probes

ZR-1 and ZR-2 probes are responsible for the detection of the elevated radiation background, which might be caused by the radiological event or hostile attack. The ZR-1 probe is a peripheral device: a gamma radiation detector and meter (with two Geiger–Müller counters). It can be installed inside or outside a collective protection facility (it adheres rigidly to the outdoor liner). The ZR-1 probe works coherently with the electronic on-board module, which records measurement results, signals the threats, and cooperates with external devices.

The ZR-2 probe is a dosimetric module that performs a similar function to the ZR-1 probe but can also detect neutrons. The difference between them is that the ZR-2 probe has a detection system based on two silicon detectors of the Si-PIN photodiode type. Both are characterized by relatively small dimensions, are resistant to mechanical and environmental factors, and show high dynamics of measured values. It has a TWI (I2C) transmission interface (serial transmission protocols).

**Figure 4:**
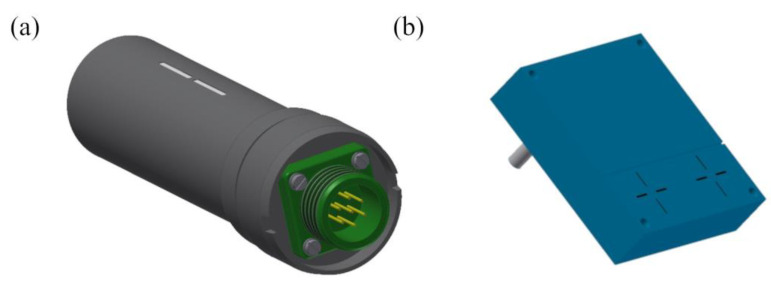
3D visualization of the (**a**) ZR-1 dosimetric probe and (**b**) ZR-2 dosimetric probe.

Both probes have a wide operating temperature range (from −30 to 60 °C) and an even greater limit range (from −50 to 70 °C). The ZR-1 and ZR-2 probes are shown in Figure 4.

The signaling device detection system can detect gamma radiation after exceeding an absorbed dose rate of 5 μGy/h.

After exceeding this permissible threshold, the signaling device provides information on the type of radiation (gamma or neutron) and activates a light and sound alarm. It is resistant to gamma radiation doses exceeding 100 Gy. The device is supplied with an internal battery (24 V ± 8 V). The conventionally true value of the measured quantity is calculated using Equation (3):

P = N_B_∙M_B_ (3)

where N_B_ is the determined calibration factor and M_B_ is the value measured by the calibrated radiometric unit.

## 3. Results

### 3.1. The Detection of CWAs

**Figure 5:**
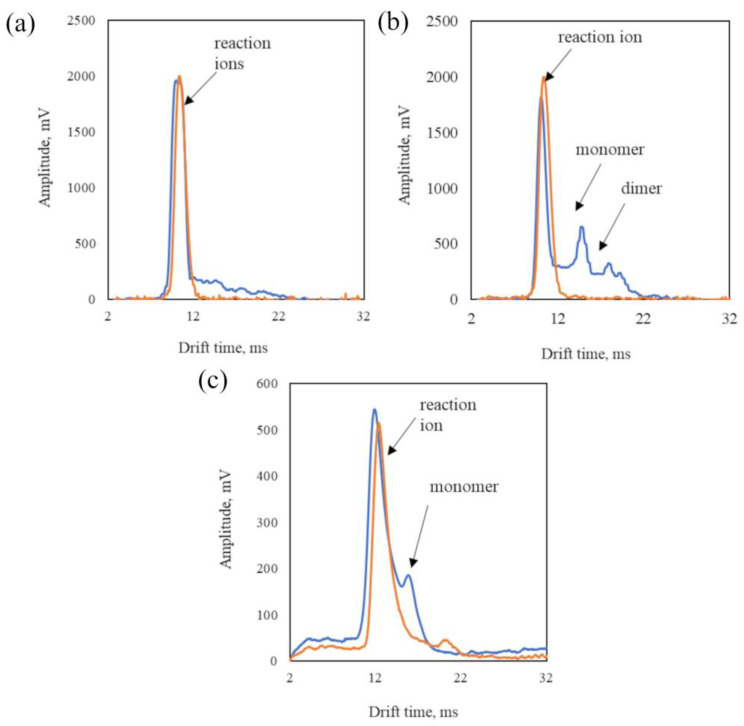
IMS spectrogram of (**a**) air analysis, (**b**) soman, and (**c**) sulfur mustard (for negative ions).

This ALERT was tested in laboratory conditions on detection. In the first step, the IMS was air-tested. The air drift time spectra are shown in Figure 5a.

Standard measurement conditions are a gas flow of 1 dm^3^/min. The temperature is 40 °C in the DT IMS chamber, but the air temperature ranges from −30 to 50 °C. The content of the gases in the air was given according to the indications of the detection limit in the Defense Standard NO-42-A221, “Equipment for detecting chemical contamination.” Technical requirements of 0.089 and 2.5 mg/m^3^ were set for the detection of soman and sulfur mustard, respectively.

ALERT can obtain drift time spectra, presented in Figure 5. The starting point for further analyses is the spectra of air, where reaction ions (H^+^) are visible (Figure 5a). Exemplary CWA spectra for soman (Figure 5b) and sulfur mustard (Figure 5c) are presented. Substances are distinguished by the position of the monomer (MH^+^) and dimer (M_2_H^+^) peaks.

After a CWA is introduced to the DT IMS, additional peaks appear with K_0_ values: 1.32 cm^2^V^−1^s^−1^ and 1.02 cm^2^V^−1^s^−1^ (±0.05). In the case of soman IMS, it ionizes, increases its temperature, and reduces the mobility of the ions formed by almost twofold. It is positively polarized, like other second-generation poisonous CWAs, such as tabun or sarin. In such a case, the signaling device generates information about the detected G-type contamination and activates the contamination alarm. Drift time spectra for sulfur mustard are shown in Figure 5c. A monomer peak of mustard gas is shown. The reaction time for threshold concentrations is 30 s for CWAs (Table 1) and 1 min for TICs.

For much higher concentrations of CWAs and TICs, the reaction time is only a few seconds.

### 3.2. Detection of Gamma Radiation

**Figure 6:**
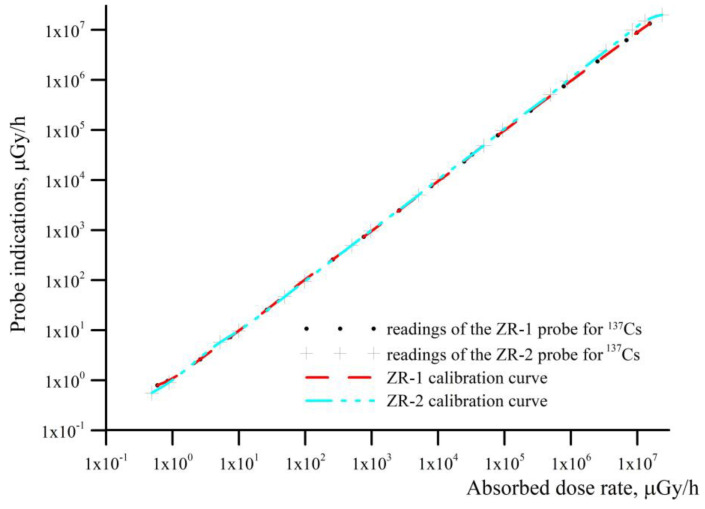
Registered dose rate of the ZR-1 and ZR-2 dosimetric probes as a function of the absorbed dose rate for the ^137^Cs isotope.

Since the detection of ionizing radiation might be carried out using ZR-1 and ZR-2 dosimetric probes, Figure 6 shows the deviations of the dose rate indicated by these probes on the calibrated reference value of the dose rate.

Both probes work very evenly, as shown in Figure 6. They perform quick and sufficient identifications of emergency radiation situations. For the ZR-1 probe, the response time to the existing threat of ionizing radiation is visible at the absorbed dose rate of 0.8 μGy/h, whereas for the ZR-2 probe, it is visible at the absorbed dose rate of 0.6 μGy/h. According to the manufacturer, Polon Alfa, the measurement accuracy of the ZR-1 and ZR-2 dosimetric probes is ±15%. After connecting the dosimetry module to the ALERT gas alarm device, it is possible to program alarm thresholds, which enables dosimetry control for both small and large doses and volumes.

The probes can work together or separately, and their measuring range is up to 100 Gy/h. The ZR-1 probe at the limit of approximately 10 mGy/h switches the Geiger–Müller counters. This is where the hysteresis loop occurs. In the ZR-2 probe, the silicon diodes switch the range at the radiation dose value of about 75 mGy/h.

**Figure 7:**
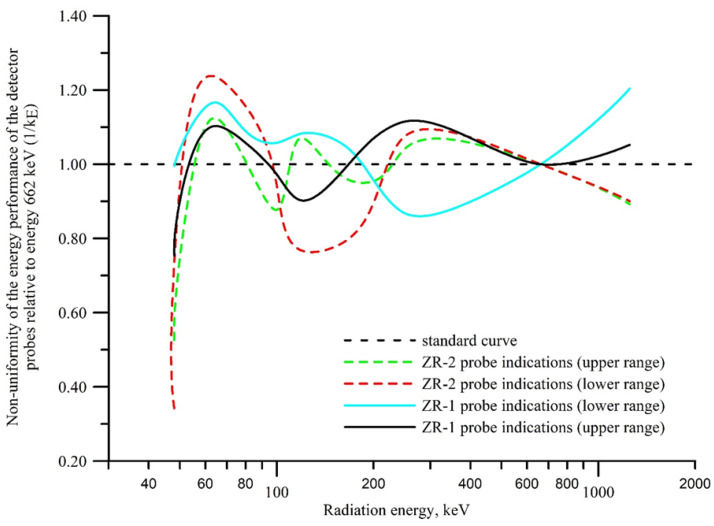
Changes in indications of the dosimetric probes: (**solid line**) the ZR-1 probes in the upper measuring range from 32 mGy/h to 16 Gy/h and the lower range from 0.5 µGy/h to 25 mGy/h; (**dashed line**) the ZR-2 probe in the upper measuring range from 75 mGy/h to 24 Gy/h and the lower measuring range from 0.4 µGy/h to 75 mGy/h.

The energy characteristics of the ZR-1 and ZR-2 dosimetric probes in the energy range from 48 to 1250 keV is shown in Figure 7.

The reference energy is ^137^Cs (662 keV). This is the value where both measuring ranges of both the ZR-1 and the ZR-2 probes meet. The probes meet military and civilian requirements because the radiation measured by them is within the energy ranges required by the standards. At the initial energy values, the detectors’ indications were significantly lower. Only at the value of approximately 63 keV did they approach the reference value. The inequality of the energy performance of both probes ranged from +1.23 to −0.77 in relation to the energy of 662 keV (^137^Cs) in the energy range from 45 to 1250 keV.

## 4. Conclusions

There are real risks of contamination from the uncontrolled release of highly toxic chemicals mainly as a result of industrial or transportation accidents. Chemical, biological, and radioactive substances can be released into the environment as a result of natural factors, such as hurricanes, floods, and earthquakes, or human factors, such as human error (accidental) and terrorist attacks (deliberate). This enforces the need for an effective real-time contamination detection system.

ALERT is a device designed mainly for monitoring chemical air contamination in collective protection facilities, both stationary and mobile, and for controlling filtering devices in such facilities. The signalizer allows for the detection and identification of CWAs and the signaling of their concentration according to programmed detection thresholds, in addition to determining the concentration level of selected TICs. ALERT can detect and measure the magnitude of doses, as well as the magnitude of surface contamination with γ and neutron radionuclides. The dosimetry module has programmable alarm thresholds, which allows for the dosimetric control of both low and high dose rates and magnitudes. The use of a special design has made the beacon suitable for use as a portable, on-board, and stationary device.

The transmission of data from reconnaissance can be carried out automatically via radio adapted for digital communication to the CDS’s contamination detection system in the form of reports (CBRN-1) or from fixed or mobile telephony. Internal batteries provide up to 2 h of continuous operation without external power. The ALERT system can be used in various types of shelters, from warships to stationary shelters, or mobile temporary shelters.

## Figures and Tables

**Table 1:** Detection threshold of particular CWAs by the IMS module.

CWAs	Detection Threshold/ Concentration Level [ppm]	CWA Type
**organophosphorus compounds such as sarin, soman, Vx, and tabun**	50	G
**blistering agents such as sulfur mustard and lewisite**	500	H

G: nerve agents; H: blister agents/vesicants.

**Table 2:** Detection threshold of chemicals from the TIC group.

TICs	Detection Threshold/ Concentration Level [ppm]
**Cl_2_**	1500
**NH_3_**	17,000

## Data Availability

Data is contained within the article.
